# The structure of sensory afferent compartments in health and disease

**DOI:** 10.1111/joa.13544

**Published:** 2021-09-15

**Authors:** Steven J. Middleton, Jimena Perez‐Sanchez, John M. Dawes

**Affiliations:** ^1^ Nuffield Department of Clinical Neurosciences University of Oxford Oxford UK

**Keywords:** central terminals, cutaneous sensory endings, DRG neurons, DRG neuron soma, neuronal compartments, neuropathic pain, node of Ranvier, pain, sensory afferents

## Abstract

Primary sensory neurons are a heterogeneous population of cells able to respond to both innocuous and noxious stimuli. Like most neurons they are highly compartmentalised, allowing them to detect, convey and transfer sensory information. These compartments include specialised sensory endings in the skin, the nodes of Ranvier in myelinated axons, the cell soma and their central terminals in the spinal cord. In this review, we will highlight the importance of these compartments to primary afferent function, describe how these structures are compromised following nerve damage and how this relates to neuropathic pain.

## Graphical abstract

Like most neurons, sensory afferents are highly compartmentalised. In this review we will discuss the structure of distinct compartments within sensory neurons, how they relate to normal sensory function and highlight anatomical changes which occur to these compartments following nerve injury and their relationship to neuropathic pain.

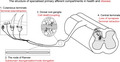

## 1 INTRODUCTION

**FIGURE 1:**
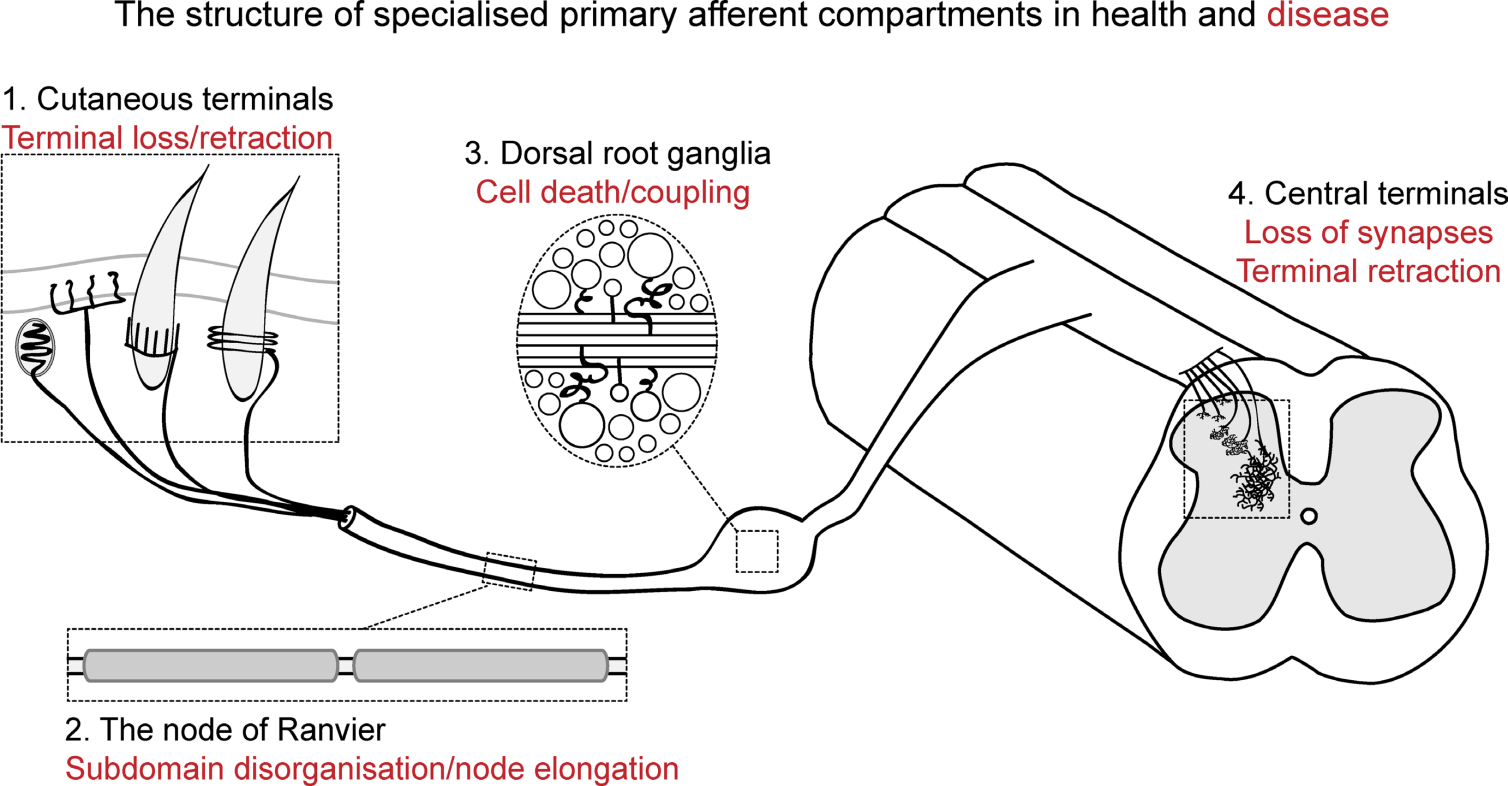
The structure of specialised primary sensory afferent compartments in health and disease

The ability to sense the environment is a vital feature which allows us to detect and react to external ques. Somatosensation relies on primary sensory afferents being able to respond to a variety of both noxious and non‐noxious triggers that signal discriminatory and/or painful events. These sensory afferents whose cell bodies reside within the dorsal root ganglia (DRG) or trigeminal ganglia are a heterogeneous population of cells able to convey information relating to distinct sensations such as touch, temperature, itch and pain. As well as understanding their basic physiology, these neurons are the focus of pre‐clinical and clinical research due to their key role in driving chronic pain, in particular pain resulting from damage or disease of the nervous system (neuropathic pain). Compared to other targets along the sensory neural axis, primary sensory afferents are considered more easily accessible for therapeutics and interventions. This is largely due to their peripheral location, the blood–nerve barrier (less limiting than the blood–brain barrier) and long‐extending axons with unique targetable compartments. Sensory afferents are highly compartmentalised and the structure of these compartments is key to normal somatosensation. In this review, we will focus on distinct neuronal compartments, including cutaneous afferents, axonal structures such as the node of Ranvier, the cell soma and central terminals, and discuss them in that order moving from the periphery to the spinal cord (Figure 1, sections 1–4). Concentrating on pre‐clinical literature, we will describe the structure of these compartments, how this relates to normal sensory function and highlight anatomical changes that occur in each of these compartments following nerve injury and their relationship to neuropathic pain.

## 2 CUTANEOUS SENSORY AFFERENT TERMINALS

The skin is a highly specialised organ able to detect and signal external stimuli. It is innervated by multiple sensory afferents which are heterogeneous in their molecular identity and expression profiles. As such, different cutaneous sensory endings express specific molecular transducers that initiate signalling in response to mechanical, thermal, chemical or purinergic stimuli. However, sensory afferents are also heterogeneous and unique in their structure, which can relate and inform on their function and role in somatosensation. Here we will highlight the structural organisation of sensory afferent terminals of the skin in health and disease. We will also discuss how sensory terminals interact with other unique cell types found in the skin.

### 2.1 Structure and function

#### 2.1.1 Longitudinal lanceolate endings

**FIGURE 2:**
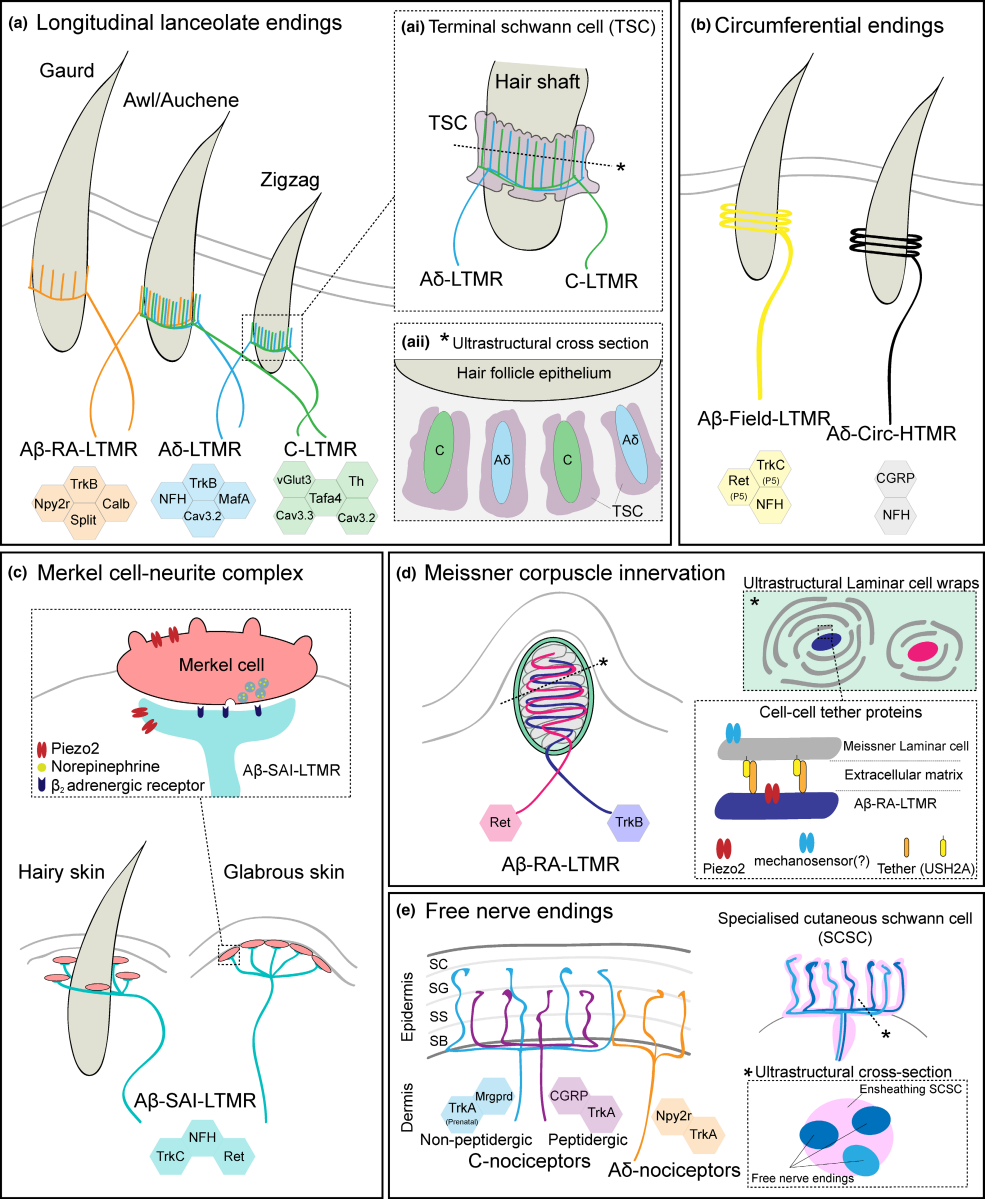
Specialised cutaneous primary afferent terminals. (a) Longitudinal lanceolate endings from molecularly distinct primary afferent populations innervate all three types of hair follicles. Guard hairs are innervated by Aβ‐RA‐LTMRs, Awl/Auchene hairs are innervated by Aβ‐RA‐LTMRs, Aδ‐LTMRs and C‐LTMRs. Zigzag hairs are innervated by Aδ‐LTMRs and C‐LTMRs. (i) Hair follicle innervation showing overleaving innervation of different terminal endings. Terminal Schwann cells (TSCs) also associated with hair follicles and longitudinal lanceolate endings. (ii) Ultrastructural depiction of a cross‐section of the hair‐TSC‐ending complex. TSCs are shown to be in close proximity and encase longitudinal lanceolate endings. (b) Two molecularly and electrophysiologically distinct populations of circumferential endings termed Aβ‐field‐LTMRs and Aδ‐Circ‐HTMRs. (c) Merkel cell–neurite complex. Merkel cells located at the dermal/epidermal border either surrounding guard hairs of hairy skin or glabrous skin. Merkel cells are mechanosensitive and form a synaptic‐like complex with Aβ‐SAI‐LTMR terminals. (d) Meissner corpuscles are located close to the epidermal/dermal border and are dually innervated by two molecular and electrophysiologically distinct populations of Aβ‐RA‐LTMRs. Ultrastructural analysis of Meissner corpuscle innervation suggests that the two distinct innervating Aβ‐RA‐LTMRs have different laminar cell wrapping patterns from Meissner corpuscle laminar cells. Meissner corpuscle laminar cells are mechanosensitive and may form extracellular tethers/tether‐like complexes with Aβ‐RA‐LTMR endings. USH2A has been identified as a candidate Meissner corpuscle protein involved in the tether‐like‐complex. (e) Free nerve endings which originate from nociceptors terminate in the epidermis. Distinct termination patterns have been observed for different nociceptor populations. Most notably non‐peptidergic nociceptors terminate most distally in the stratum granulosum (SG). Nociceptors have been shown to associate closely with specialised cutaneous Schwann cells (SCSCs). Ultrastructural cross‐section of free‐nerve endings and SCSCs illustrate that SCSCs sheath free nerve endings

Longitudinal lanceolate endings are sensory afferent endings that surround hair follicles, giving rise to ‘finger‐like’ structures that are associated with all three types of hair follicles, (zigzag, awl/Auchene and guard). In mice, longitudinal lanceolate endings are the terminals of Aβ‐ rapidly adapting (RA) low threshold mechanoreceptors (LTMRs), Aδ‐LTMRs and C‐LTMRs (Figure 2a) (Bernal Sierra et al., 2017; François et al., 2015; Li & Ginty, 2014; Li et al., 2011; Luo et al., 2009). All three types of mouse hair follicles are innervated by one or more lanceolate endings. Zigzag hairs are innervated by C‐LTMRs and Aδ‐LTMRs. Awl/Auchene hairs are innervated by C‐LTMRs, Aδ‐LTMRs and Aβ‐rapidly adapting (RA) LTMRs and guard hairs are innervated by Aβ RA‐LTMRs (Li & Ginty, 2014; Li et al., 2011). Longitudinal lanceolate endings from different fibre types innervating the same follicle show an interdigitated organisation (Li et al., 2011). Each fibre type can be distinguished by histochemical markers or genetic labelling (for some molecular markers, see Figure 2) and there is evidence that transcription factors, such as Runx1 in the case of C‐LMTRs, can control lanceolate ending morphology (Lou et al., 2013). Hair follicles innervated by Aδ‐LTMRs can be directionally sensitive (Rutlin et al., 2014; Walcher et al., 2018). This attribute is thought to arise from polarised Aδ‐LTMRs surrounding the hair follicle, driven by epidermal brain‐derived neurotrophic factor (BDNF) expression patterns (Rutlin et al., 2014). This may extend to other lanceolate endings but that has not yet been fully resolved for all fibre types. Longitudinal lanceolate endings are also associated with accessory terminal Schwann cells (TSC) which encapsulate the longitudinal endings (Figure 2a(i)). The development and maintenance of this mechanoreceptor/glia structural complex are dependent on sensory neuron‐derived glutamate (Woo et al., 2012), and lanceolate endings can continuously release glutamate from synaptic‐like vesicles which are thought to modulate vesicle recycling and afferent activity (Banks et al., 2013). In addition, the presence of TSCs is required to maintain lanceolate ending structure and morphology. Genetic ablation of TSCs resulted in de‐innervation of lanceolate endings (Li & Ginty, 2014). However, other than the obvious de‐innervation, the impact on afferent function in behaving animals was not explored. Ultra‐structural analysis revealed that a single terminal Schwann cell encapsulates multiple hair follicle endings (Figure 2a(ii)) and identified the presence of intercellular processes that resemble tether‐like proteins, physically linking epithelial cells of hair follicles to lanceolate endings and TSCs (Li & Ginty, 2014). It has been proposed that tether proteins may facilitate the mechanotransduction of mechanoreceptors in the skin (Hu et al., 2010). Using ultrastructural imaging, extracellular tether‐like proteins appear to link sensory neurons and fibroblasts when in co‐culture, as well as sensory neuron neurites to laminin substrates. Selective protease treatments (subtilisin) abolished both the presence of tether‐like structures and the rapidly adapting mechanosensitive currents (elicited from neurite stimulation). The mechanosensitivity of these receptors in the skin was also shown to require a subtilisin‐sensitive protein (Hu et al., 2010). It is unclear how tether proteins may modulate mechanotransduction, and if they directly gate mechanosensitive ion channels or if they play a part in other ways such as contributing to membrane stiffness.

#### 2.1.2 Circumferential endings

Circumferential endings are terminal endings that circumferentially wrap around hair follicles (Figure 2b). Notably, these endings are neurofilament‐heavy chain positive. One population of these endings is thought to be the endings of Aβ‐Field LTMRs. Aβ‐Field LTMRs are slowly adapting mechanoreceptors that have large expansive receptive fields (Bai et al., 2015). They are thought to be responsible for the detection of skin stroking, but not hair deflection (Bai et al., 2015). A second population of afferents that terminate as circumferential endings is a sub‐population of Aδ‐high threshold mechanoreceptors (HTMRs) that have been termed Circ‐HTMRs (Ghitani et al., 2017). Circ‐HTMRs have spatially organised receptive fields that lend them to respond to a single hair pull and optogenetic activation of this population evokes avoidance behaviour and guarding. Collectively, this suggests this population of afferents are nociceptive and mediate the fast response associated with hair‐pull (Ghitani et al., 2017).

#### 2.1.3 Merkel cell–neurite complex

Merkel cells are oval‐shaped cells located at the basal layer of the epidermis (Figure 2c). Merkel cells are predominantly found in touch domes of glabrous skin, however, they are also associated with guard hair follicles on hairy skin. Merkel cells form protrusions that anchor them to surrounding structures and are associated with the dense endings of Aβ‐slowly adapting type 1 (SAI) LTMRs (Iggo & Muir, 1969; Woodbury & Koerber, 2007). A single Aβ‐SAI‐LTMR terminates with multiple dense endings, each associating with a Merkel cell and forming a Merkel cell–neurite complex. The close association of Aβ‐SAI‐LTMR endings and Merkel cells are reminiscent of synaptic or synaptic‐like structures (Hartschuh & Weihe, 1980; Mihara et al., 1979). Indeed, Merkel cells express synaptic machinery (Haeberle et al., 2004). This has led to evidence that Merkel cells form glutamatergic or serotonergic synaptic contacts with Aβ‐SAI‐LTMRs (Chang et al., 2016; Fagan & Cahusac, 2001; He et al., 2003; Hitchcock et al., 2004; Press et al., 2010). However, this was challenged by Hoffman et al. (2018) who systematically investigated functional transmission at the Merkel cell–neurite complex. This work identified SNARE‐dependent adrenergic synapses that directly activate Aβ‐SAI‐LTMRs (Figure 2c) (Hoffman et al., 2018). Merkel cells are themselves mechanosensitive, require the mechanosensitive ion channel Piezo 2 and fine‐tune light touch sensation (Maricich et al., 2009; 2012; Maksimovic et al., 2014; Woo et al., 2014). Recently, Merkel cells have also been implicated in transmitting the conversion of touch stimuli to itch, particularly in the context of alloknesis (Feng et al., 2018).

While Merkel cells make up approximately 3%–6% of skin cells, keratinocytes are by far more abundant. Their abundance and physical proximity to sensory afferents have led to their investigation in sensory function and pain (for review, see Moehring, Halder et al., 2018). In particular, keratinocytes have been shown to modulate both innocuous and noxious mechanosensation via an ATP‐P2X4 signalling mechanism (Moehring, Cowie et al., 2018).

#### 2.1.4 Meissner corpuscles

Meissner corpuscles consist of an axon (or axons) and specialised Schwann cells (also termed laminar cells), contained within a capsule structure. Meissner corpuscles are primarily associated with Aβ‐RA‐LTMRs (Figure 2d). Meissner corpuscle laminar cells surround and encompass the innervating sensory afferent. In rodents, Aβ‐RA‐LTMR innervating Meissner corpuscles are predominantly located on the glabrous skin (Luo et al., 2009) and are exquisitely more sensitive on rodent forepaws (Walcher et al., 2018). They are located at the dermal papillae and are held in place by collagen fibres. Meissner corpuscles can be identified by their S100 expression and the unique zig‐zag organisation of the innervating Aβ‐RA‐LTMRs. A single Meissner corpuscle can be innervated by up to three myelinated fibres and Aβ‐RA‐LTMRs can branch extensively and innervate multiple Meissner corpuscles (Cauna & Ross, 1960; Jänig, 1971; Neubarth et al., 2020). In particular, a recent study used elegant genetic labelling of two non‐overlapping primary afferent populations and showed that Meissner corpuscles are dually innervated by multiple mechanoreceptive fibres that have distinct physiological properties (Figure 2d) (Neubarth et al., 2020). In addition, there is some evidence that some Meissner corpuscles may also be closely associated with afferents that have nociceptive characteristics (Ishida‐Yamamoto et al., 1988; Johansson et al., 1999; Paré et al., 2001). However, the role of these innervating nociceptive afferents has yet to be fully characterised on a functional level.

Usually, the sensory afferent is the key focus when studying Aβ‐RA‐LTMR mechanoreception (Hao et al., 2013; Heidenreich et al., 2012). However, recent studies have begun to focus on Meissner corpuscles more directly. Meissner corpuscle protein USH2A has been implicated as necessary for tuning the vibration sensitivity of Aβ RA‐LTMRs in mice and humans (Schwaller et al., 2021). Structurally, USH2A has a large extracellular domain making it a possible candidate for the tether‐like protein/protein complex thought to facilitate the detection of fine‐grained tactile objects (Figure 2d) (Hu et al., 2010; Schwaller et al., 2021). It is still unclear if Meissner corpuscle Aβ RA‐LTMRs communicate through physical linkers or if they also have synaptic‐like contacts. It has been shown that Meissner corpuscle laminar cells are themselves mechanosensitive, excitable and can generate current injection evoked action potentials which are dependent on R‐type voltage‐gated calcium channel Cav2.3 (Nikolaev et al., 2020). This is indicative that synaptic‐like contacts may exist to relay laminar cell mechanosensitive signalling to Aβ RA‐LTMRs. Indeed, there is some evidence that Meissner corpuscle laminar cells contain dense synaptic‐like vesicles in close proximity to Aβ RA‐LTMR endings (Nikolaev et al., 2020).

#### 2.1.5 Pacinian corpuscles and Ruffini endings

In humans, Pacinian corpuscles are extremely sensitive and classically encode high‐frequency vibration stimuli (Johansson et al., 1982). Pacinian corpuscles are densely located in the hand and fingers and structurally they are oval‐shaped with interdigitating laminar cells (Halata, 1977). Each Pacinian corpuscle is thought to be innervated by a single Aβ RA‐II LTMR. While they have been identified in human skin, they have not been identified in mouse skin. In mice, Pacinian corpuscles are primarily associated with joints and the periosteum of bones. Due to their relative inaccessibility, the study of Pacinian corpuscles in rodents is often overlooked. However, it has been demonstrated that the development of Pacinian corpuscles is critically dependent on Ret signalling (Fleming et al., 2016; Luo et al., 2009) and the transcription factor c‐Maf (Wende et al., 2012). It has been recently shown that Pacinian corpuscle laminar cells are mechanosensitive (Nikolaev et al., 2020). Additionally, a recent study used a novel *ex vivo* periosteum/bone‐nerve preparation to demonstrate that mouse Pacinian corpuscles encode high‐frequency vibrations (Schwaller et al., 2021).

Similarly, evidence for the existence of Ruffini endings in mouse skin is lacking and debated. Ruffini endings resemble Golgi end organs and are thought to encode stretch. It is hypothesised that stretch responsive SA‐II‐LTMRs identified in humans innervate Ruffini endings, however, direct evidence of this is lacking (Chambers et al., 1972). SA‐II LTMR responses have only relatively recently been identified in rodent skin (Wellnitz et al., 2010) and the structure they terminate is unclear. However, there is evidence that in mice, Ruffini‐like endings innervate periodontal ligaments (Matsuo et al., 2002; Rahman et al., 2011).

#### 2.1.6 Free nerve endings

Free nerve endings are the common terminal endings of most nociceptive afferents. These endings are considered to be the most distally located of all cutaneous sensory endings. Free nerve endings reach the sub‐epidermal border, where they branch first horizontally followed by extensive vertical branching into the epidermis (Figure 2e). C‐fibre nociceptors that terminate as free nerve endings can be dived into two major subpopulations, peptidergic (CGRP, SP positive) and non‐peptidergic (IB4 binding or MrgprD, P2X3R positive) afferents. Interestingly, free nerve endings from these two C‐fibre populations are spatially segregated, with non‐peptidergic afferents terminating in the stratum granulosum of the epidermis (Figure 2e) (Zylka et al., 2005). Differential terminal organisation of different C‐fibre populations supports the idea that different subpopulations play unique roles in pain processing. Many studies have used inactivation or ablation techniques to interrogate this question. It is thought that peptidergic afferents encode heat pain and non‐peptidergic afferents encode mechanical pain (Cavanaugh et al., 2009; McCoy et al., 2013). However, depending on the strategy used, this has been both confirmed and challenged (Cowie et al., 2018; Ferrini et al., 2020; Fitzgerald & Woolf, 1982; Karai et al., 2004; Mishra & Hoon, 2010; Neubert et al., 2003; Nocchi et al., 2019; Pinto et al., 2019; Tarpley et al., 2004; Vulchanova et al., 2001; Yaksh et al., 1979). In humans, congenital insensitivity to pain can arise from rare Mendelian genetic disorders. A number of these genetic disorders lead to the failure of nociceptors to develop and a subsequent lack of epidermal free nerve endings (Drissi et al., 2020). This exemplifies the critical role of epidermal C‐fibres in human nociception.

Most thinly myelinated Aδ‐nociceptors also terminate in the epidermis as free nerve endings (Figure 2e), however, this is subpopulation dependent (Arcourt et al., 2017; Ghitani et al., 2017). While there is physiological evidence for cutaneous Aβ‐nociceptors (Djouhri & Lawson, 2004; Nagi et al., 2019), their cutaneous terminal morphology has yet to be resolved.

It is widely accepted that free nerve endings originate from nociceptors and signal aversive and painful stimuli (Basbaum et al., 2009). However, free nerve endings may not act alone. Other cutaneous structures have been shown to associate closely with free nerve endings and contribute to their role in pain processing. Recently, a study identified the molecular and functional identity of specialised cutaneous Schwann cells (Abdo et al., 2019). These specialised cutaneous Schwann cells were found to form a network at the sub‐epidermal border, where they extend radial processes into the epidermis. It was discovered that free nerve terminals of nociceptive afferents were ensheathed by the cutaneous Schwann cell and their processes (Figure 2e) (Abdo et al., 2019). The term glio–axonal complex was coined to describe this relationship between the two cell types. These specialised Schwann cells were also shown to be directly mechanosensitive and their optogenetic activation initiated pain‐related behaviours and determined mechanical thresholds of mice (Abdo et al., 2019). Further analysis has demonstrated that in the mouse, there is an interdependence of the nerve and the nociceptive Schwann cell, and loss of intraepidermal Schwann cells can result in neuropathic‐like pain behaviours (Rinwa et al., 2021). These specialised Schwann cells have now also been identified in human skin (Rinwa et al., 2021). The identity and structure of this novel cell type will undoubtedly lead to new and important discoveries for pain biology.

### 2.2 Changes in cutaneous innervation following nerve injury or disease

Sensory fibre degeneration and regeneration often accompany neural injury and have been well studied in rodents (Dubový et al., 2018; Navarro, 2016). In humans, studying degeneration and regeneration is more difficult, but advances are being made (Scheib & Höke, 2013). In humans and rodents, injury or disease can lead to degeneration or retraction of sensory afferents in the skin. In particular, it has been reported that people living with neuropathic pain have a reduced intraepidermal nerve fibre density (IENFD). Small fibre involvement has been shown for many different painful peripheral neuropathies such a painful diabetic polyneuropathy, entrapment neuropathy, Guillain‐Barré syndrome, complex regional pain syndrome, and more (Martinez et al., 2010; Pittenger et al., 2004; Rasmussen et al., 2018; Schmid et al., 2014; Themistocleous et al., 2016). IENFD is also reduced in rodent models of neuropathic pain, such as nerve injury/lesion, high‐fat diet diabetic neuropathy, pancreatic β‐cell ablation model (STZ) of diabetic neuropathy and chemotherapy‐induced neuropathy (Duraku et al., 2013; Hedstrom et al., 2014; Jayaraj et al., 2018; Lindenlaub & Sommer, 2002; Ma & Bisby, 2000; Wozniak et al., 2018). These findings suggest that free nerve endings in particular are vulnerable afferents and are susceptible to retraction or degeneration following injury or disease and consequently quantification of IENFD has arisen as a useful and relevant measure of neuropathy in patients. Patients with polyneuropathies or demyelinating neuropathies can also show a loss of dermal myelinated fibres and changes in nodal architecture (Doppler et al., 2012; Doppler et al., 2013). Studies focused on entrapment neuropathies, such as carpel tunnel syndrome, have debated the loss/involvement of myelinated fibres, which may reflect the difference in skin biopsy sites used for analysis (Provitera et al., 2020; Schmid et al., 2014). In addition, degeneration of sensory afferents and reductions in IENFDs may also reflect the heterogeneity of many neuropathies that often display a mix of loss and gain of function sensory characteristics.

It remains challenging to identify the molecular mechanisms that underpin these innervation changes and how this directly links to the pain experienced by patients. However, pre‐clinical neuropathic pain models have demonstrated that IENFD is reduced and sensitivity to noxious stimuli increases. Over time recovery of pain‐related behaviours is accompanied by IENFD reinnervation (Lindenlaub & Sommer, 2002). However, it has been observed that different sensory afferents may have unique reinnervation patterns/time courses (Duraku et al., 2013; Peleshok & Ribeiro‐Da‐Silva, 2011). In humans, longitudinal skin biopsy sampling in patient cohorts can aide this investigation (Hahn et al., 2006; Petersen et al., 2010; Polydefkis et al., 2004; Rajan et al., 2003). In particular, there has been recent histological evidence that surgical interventions in humans can promote IENF regeneration. Free nerve endings were measured in a cohort of carpel tunnel syndrome (CTS) patients pre‐ and post‐decompression surgery (Baskozos et al., 2020). Interestingly, patient's IENFD improved following surgery, and the level of nerve fibre regeneration positively correlated with symptom improvement, including pain (Baskozos et al., 2020). This study also identified a molecular signature for nerve regeneration in the skin using RNA sequencing. The authors focus on the most differentially regulated gene ADCYAP1 that encodes PACAP which facilitated neurite outgrowth when given to human‐induced pluripotent stem cell‐derived sensory neurons (Baskozos et al., 2020). This work contributed to the breadth of literature that growth factors may have therapeutic promise for improving the regeneration of peripheral nerves following injury (McGregor & English, 2019; Mickle et al., 2019).

There are also clinical and preclinical data suggesting that regeneration can be improved in an activity‐dependent manner (McGregor & English, 2019; Udina et al., 2011). In particular, the advances in chemo/optogenetic tools have enabled researchers to precisely control neuronal activity and study regeneration (Park et al., 2015; Ward et al., 2018; Ward et al., 2016). Jayaraj et al. (2018) used the high‐fat diet model of diabetic neuropathy in mice and demonstrated that IENFD was reduced and mice displayed neuropathic pain‐like behaviours. They showed that long‐term chemogenetic inhibition of nociceptors prevented both small fibre degeneration and neuropathic hypersensitivity (Jayaraj et al., 2018).

Taken together, loss of IENFD is associated with neuropathic pain and reinnervation is correlated with symptom improvement. This is an interesting, but perhaps counter‐intuitive, observation when considering evoked neuropathic pain in humans and rodents. However, the largest clinical complaint is ongoing pain, which may arise from damaged sensory afferents that then develop a spontaneous activity. This would support the idea that loss of IENFD correlates with neuropathic pain symptoms. More work is required to understand and pinpoint why a structural loss of epidermal innervation is associated with neuropathic pain development. Indeed, structural changes following injury are dependent on many mechanisms which likely include growth factors and ongoing activity of primary afferents which may offer new avenues for pain treatment.

## 3 THE NODE OF RANVIER

**FIGURE 3:**
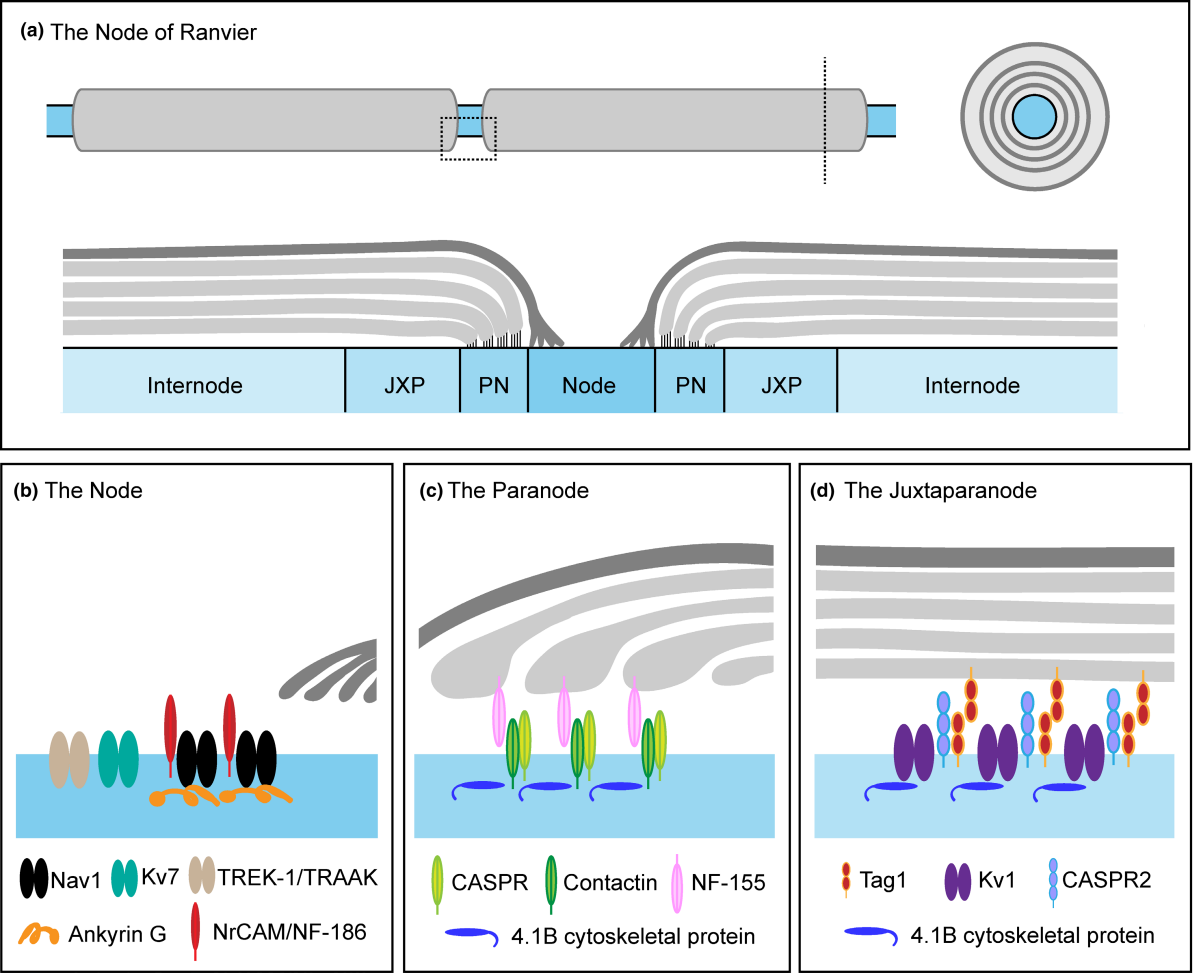
The node of Ranvier. (a) The node of Ranvier is organised into specific sub‐domains through axoglial interactions. (b) The node occurs at gaps between myelinated Schwann cells, exposing the axon to the extracellular environment, and is crucial for action potential regeneration. Schwann cell microvilli contact the node. The node is characterised by a high density of VGSCs which are maintained by cell adhesion molecules such as NF‐186, MrCAM, Amkyrin‐G and βIV spectrin. (c) The paranode flanks the node and is defined by the formation of axoglial junctions. At the paranode, the cell adhesion molecules Contactin, Caspr and NF‐155 facilitate the interaction between the axon and Schwann cell. (d) The juxtaparanode is located underneath compact myelin and is characterised by the presence of Kv1 channels which require the cell adhesion molecules Caspr2 and Tag1/Contactin‐2 and the cytoskeletal protein 4.1B for their localisation

Following detection, propagation of sensory information to the CNS occurs along the axon. This will include the unmyelinated axons of C‐fibres that are grouped together in Remak bundles formed by non‐myelinating Schwann cells. These axons constitute the majority of nociceptors; here, however, we will focus on one well‐defined neuronal compartment, the node of Ranvier (Figure 3a), present in myelinated sensory neurons, which will include Aβ/δ nociceptors as well as LTMRs. This structure is highly organised; it is key to saltatory conduction allowing for fast and efficient signal propagation and its organisation has been well documented (Rasband & Peles, 2016; Salzer et al., 2008). Here we will give an overview of the structure of the node and highlight changes that occur following nerve injury and their possible role in regulating neuropathic pain.

### 3.1 Structure and function

The node of Ranvier is organised into sub‐domains which include the node, paranode, juxtaparanode and internode. The node, which occurs between gaps of the myelinated Schwann cells, is ~1 μm in length and exposes the axon to the extracellular environment. It is characterised by a high density of voltage‐gated sodium channels (VGSCs) which are essential for the regeneration of action potentials. The structure of the node is maintained through complex molecular interactions (Figure 3a‐d). Schwann cell microvilli contact the node (Ichimura & Ellisman, 1991) and signal to nodal cell adhesion molecules, such as Neurofascin (NF)‐186 and NrCAM (neuronal cell adhesion molecule) which recruit the cytoskeletal proteins ankyrin‐G and βIV spectrin for clustering and maintenance of VGSCs within this domain (Figure 3b) (Amor et al., 2014; Dzhashiashvili et al., 2007; Feinberg et al., 2010; Koticha et al., 2006). In sensory neurons, the predominant sodium channel sub‐unit found at the node is Nav1.6 (Caldwell et al., 2000). Other VGSCs are also expressed in the node, including Nav1.7 and Nav1.9, found predominantly in myelinated nociceptors (Black et al., 2012; Fjell et al., 2000) as well as some evidence to suggest Nav1.8 expression (Pryce et al., 2019) and of course, these channels are required for normal sensory transmission and pain sensation (Bennett et al., 2019). The node also contains potassium channels. For example, the voltage‐gated potassium channels (VGKCs) Kv7.2 and 7.3 sub‐units which are thought to prevent repetitive firing (Pan et al., 2006), and two‐pore potassium channels such as TREK‐1 and TRAAK, where direct patching of the node suggests a role for these channels in action potential regeneration, high‐speed conduction and normal sensory behaviour in mice (Kanda et al., 2019). The paranode flanks the node and is structurally defined by the formation of junctions between the axon and the glia cell which appear as a series of transverse bands (Figure 3c). This axoglial junction acts as a barrier to avoid lateral movement of ion channels, maintaining nodal domains and protecting nodal currents for reliable saltatory conduction (Rosenbluth, 2009). The interaction of the axon and Schwann cell is facilitated by cell adhesion molecules such as Contactin, Caspr and NF‐155. Genetic knockout models of these proteins disrupt paranodal structure; there is a loss of axoglial junctions, a widening of the space between the axon and Schwann cell and mislocalisation of ion channels which impact axonal function as evidenced by reduced conduction velocities (Pillai et al., 2009; Bhat et al., 2001; Boyle et al., 2001). Interestingly, genetic mutations of NF‐155 in humans disrupt the structural integrity of the paranodal junctions and result in loss of reaction to pain and touch (Smigiel et al., 2018). The juxtaparanode is characterised by the expression of VGKCs, in particular Kv1.1 and 1.2 which require the cell adhesion molecules Caspr2 and Contactin‐2 and the cytoskeletal protein 4.1B for their correct localisation (Poliak et al., 2003) (Figure 3d). In the naïve state, it is generally considered that the Juxtaparanode Kv1 channels have limited impact on the propagation of sensory information presumably because of their isolation from the node due to the paranodal junctions. For example, preventing their localisation at the juxtaparanode or using pharmacological block does not alter conduction velocities in myelinated fibres (Bostock et al., 1981; Poliak et al., 2003). The internode, located under the compact myelin, represents the largest of these domains roughly 1 mm in length (Abe et al., 2004). Interactions between the axon and Schwann cell are mediated by a different set of different cell adhesion molecules at the internode (e.g., Nectin‐like (Necl) proteins, Necl‐1, Necl‐2 and Necl‐4) (Maurel et al., 2007) and there is a functional relationship between internode length and conduction velocity (Wu et al., 2012). The node is therefore an important axonal structure needed for proper transmission of sensory information including pain. However, its complex organisation makes this compartment vulnerable to dysfunction if this structural organisation is compromised (e.g., following nerve damage) and due to its clear role in regulating action potential generation, suggests that this dysfunction could contribute to neuropathic pain.

### 3.2 Changes to nodal structure following nerve injury or disease

As well as C‐fibre nociceptors, there is good evidence to show that A‐fibres are also important in neuropathic pain (Campbell et al., 1988; Dhandapani et al., 2018; Xu et al., 2015) and dysfunction of the node may be an important contributor. Peripheral nerve injury results in the formation of atypical nodal structures such as heminodes, split nodes and nodes lacking paranodal or juxtaparanodal structures. In an infraorbital nerve lesion model, around 50% of nodes were atypical and in these or in typical nodal structures, there is a significant decrease in the paranodal protein Caspr and an increase in Nav1.6 accumulation (Henry et al., 2006; 2007). This accumulation might result from overexpression of Ankyrin‐G at the site of injury (Kretschmer et al., 2002) and is correlated with neuropathic pain behaviour (Henry et al., 2006). One consequence of an axonal injury is the potential formation of neuromas, which are enlargements of the nerve that develop due to unorganised nerve fibre growth as the nerve attempts to heal as well as the growth of non‐neural tissue. They can be nerve‐end neuromas that occur due to complete nerve transection such as following limb amputation (Buch et al., 2020) or occur along the axon such as in Morton's neuroma (Mak, Chowdhury & Johnson, 2021). These pathological axonal structures are considered a key site of ectopic activity and neuropathic pain, and surgical removal can provide pain relief (Poppler et al., 2018). The contribution of neuromas to neuropathic pain most likely occurs due to the uncontrolled and undirected fibre growth as well as underlying molecular changes. These include the sprouting of unmyelinated C‐fibres (Devor & Wall, 1976) and the accumulation of sodium channels (Black et al., 2008) but also the formation of disorganised nodes. For example, similar to partial nerve injury models, nodal disorganisation is also seen in models of neuroma, where Caspr and Caspr2 are redistributed to the juxtaparanode and paranode, respectively, although ultrastructure analysis suggests a minimal impact on paranodal junction integrity (Calvo et al., 2016). In this same model, Kv1.1 and 1.2 channels are lost from the juxtaparanode as increased pain sensitivity and spontaneous primary afferent activity develops. However, other Kv1 channels (Kv1.4, 1.6) are upregulated and their increased expression in the node is correlated with reduced spontaneous activity and pain sensitivity (Calvo et al., 2016). Similar findings have also been reported for Kv7.2 (Roza, Castillejo & Lopez‐García, 2011). This injury‐induced nodal accumulation is intriguing particularly since a number of studies have shown that certain VGKCs, including Kv7.2 and some Kv1 channels, are consistently down‐regulated in nerve injury models (Du & Gamper, 2013). This may be explained somewhat by the disconnect between mRNA levels in the DRG and protein expression in the distal nerve. However, these studies do suggest there is a fine balance of VGKC expression in sensory neurons, one which strongly regulates excitability and that in some circumstances, natural overexpression of potassium channels may act as a compensatory mechanism to combat hyperexcitability caused by nodal disorganisation following nerve injury. Furthermore, nodes of Ranvier can form *de novo* in neuromas due to the accumulation of Nav1.6 (Chen et al., 2018). Genetic removal of Nav1.6 in mice prevents the formation of these nodes, reduces neuronal excitability and attenuates neuropathic pain in the spared nerve injury (SNI) model (Chen et al., 2018), implicating the node as a key structure in driving neuropathic pain.

Interestingly, immune‐mediated neuropathies such as Guillain‐Barré syndrome (GBS) and chronic inflammatory demyelinating neuropathy (CIPD), where neuropathic pain is common, are associated with antibodies which target nodal proteins and pain in these patients can be reversed with immunotherapy suggesting that these antibodies are pathogenic (Dawes & Bennett, 2020). Analysis of nerve or skin biopsies from patients with anti‐Contactin or Capsr antibodies, lack evidence of demyelination, but instead demonstrate changes in nodal structure, which are associated with deficits in nerve conduction studies (Doppler et al., 2015, 2016). Depending on the IgG subtype, these antibodies may directly disrupt the function of these structural proteins or activate complement, both of which can lead to disruption of node integrity. Treatments that reduce these antibodies can normalise nerve function and relieve pain (Doppler et al., 2016). Ultrastructure analysis of nodes in patients with NF‐155 antibodies shows clear structural changes including loss of axoglial junctions and widening of the space between the Schwann cell and axon (Koike et al., 2017; Vallat et al., 2017). Antibodies against juxtaparanode proteins such as Caspr2 are found in patients with a range of neurological conditions including Neuromyotonia and neuropathic pain. However, nerve conduction studies are generally normal in these patients and passive transfer models suggest that these antibodies do not target the node *in vivo* (Dawes et al., 2018). In addition to these immune‐mediated neuropathies, analysis of nodes in peripheral nerve or skin from patients with other types of neuropathies (e.g. hereditary, entrapment or poly‐neuropathies), also show disruption of nodal structure. Charcot‐Marie‐Tooth (CMT) disorder is a hereditary peripheral neuropathy associated with deficits in motor and sensory function. Loss of sensation is a common feature of neuropathy, but can also be coupled with ‘positive’ symptoms such as ongoing pain, a key feature of neuropathic pain. Interestingly, in some types of CMT, for example CMT1A, patients have ongoing pain as well as disorganisation of nodal domains including mislocalisation of Capsr and Kv1 channels (Laurà et al., 2014; Li et al., 2005). Aberrant redistribution of paranodal or juxtaparanodal proteins such as Caspr or Kv1 channels is also seen in nerve biopsies from Morton's neuroma patients and from skin analysis in patients with diabetic neuropathy (Calvo et al., 2016; Doppler et al., 2017). Furthermore, node elongation (node >6.1 μm) is a feature common across different types of neuropathies associated with neuropathic pain (Doppler et al., 2012; Doppler et al., 2013). In patients with diabetic neuropathy or carpal tunnel syndrome (CTS), there is an increase in the number of elongated nodes in skin samples compared with healthy controls (Doppler et al., 2017; Schmid et al., 2014). It is not completely clear as to exactly what this structural change in the node means in terms of sensory function and neuropathic pain. Elongated nodes are also found in diabetic patients without overt signs of neuropathy, and in CTS patients, elongated nodes negatively correlate with disease severity (including pain), suggesting that elongation may serve as a mechanism to maintain normal nerve function in the face of nerve damage (Doppler et al., 2017; Schmid et al., 2014). This idea was supported in a follow‐up study showing that following surgery, which is commonly associated with reduced neuropathic pain, CTS patients had more elongated nodes compared with pre‐surgery levels or healthy controls (Baskozos et al., 2020). Overall, these findings show that changes in nodal structure occur as a result of nerve damage, are associated with neuropathic pain both in pre‐clinical models and patients, and these changes are not only pathogenic but can also be compensatory suggesting the node as a key compartment contributing to sensory dysfunction in situations of nerve damage.

## 4 THE CELL BODIES OF DRG NEURONS

The cell bodies of primary sensory neurons reside within the DRG and express a variety of molecules that have been used to define neuronal sub‐populations. These neuronal somas are grouped in close proximity to each other, separated by connective tissue and there may be some crude somatotopical organisation (Puigdellívol‐Sánchez et al., 1998) although this is not well defined (Kim et al., 2016). Due to the pseudounipolar structure of DRG neurons, the cell soma is taken off the line of action potential propagation, and therefore, it is generally considered this compartment does not directly influence the transmission of sensory information. However, certain anatomical features of the cell soma may have an impact on conduction and structural changes in the DRG following nerve injury suggest this compartment might be an important contributor to neuropathic pain development.

### 4.1 Structure and function

**FIGURE 4:**
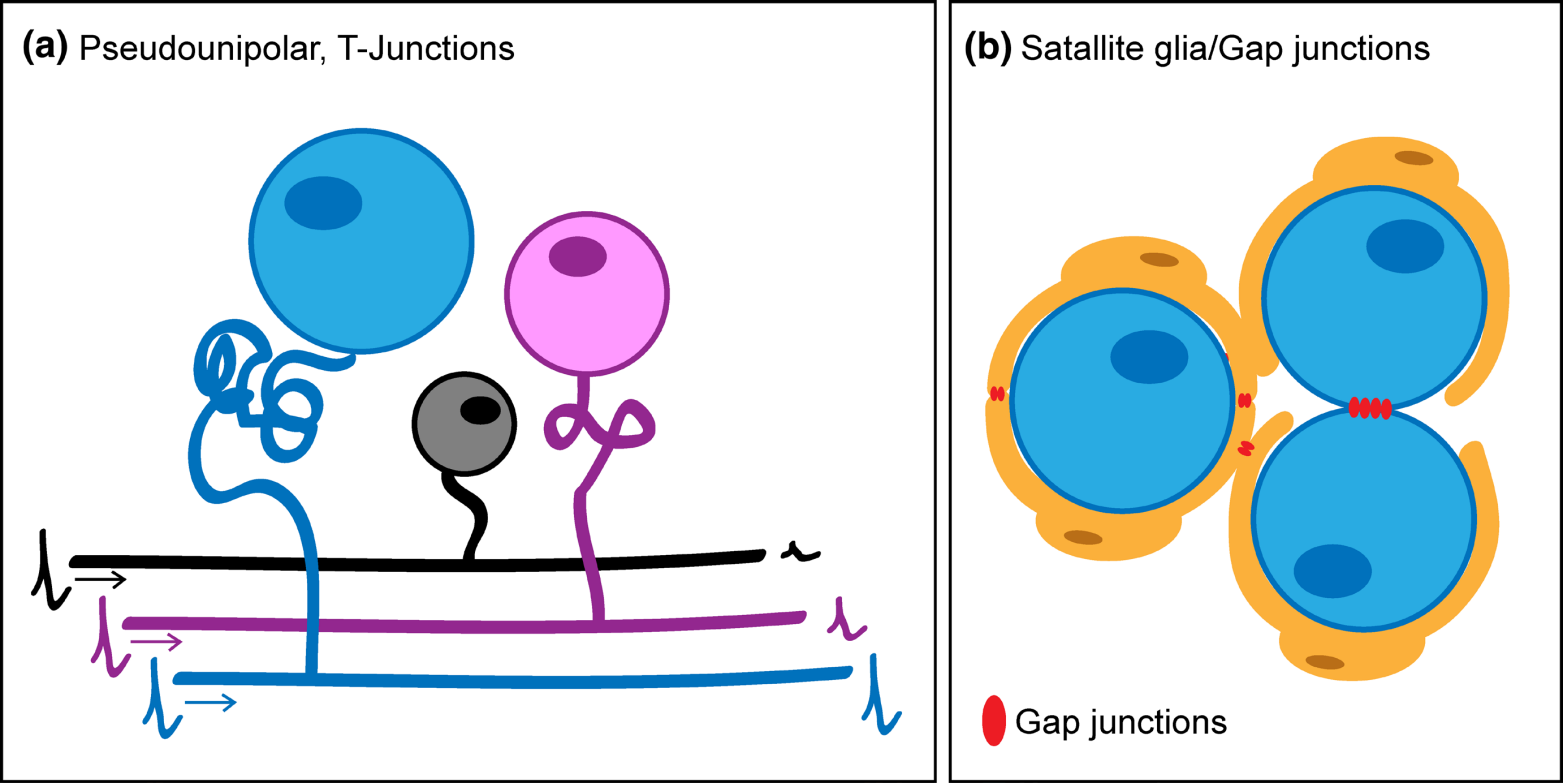
DRG neuron soma. (a) The soma of DRG neurons in mice can be defined as large (>35 μm in diameter, blue), medium (25–35 μm, pink) or small (<25 μm, black) and soma size is related to the myelination state of the neuron. Due to their psuedounipolar structure, DRG neurons have a stem axon which leaves the soma and bifurcates at the T junction. It is thought that the length of this stem axon is greater in neurons with a larger cell body, limiting its impact on conduction. DRG neurons with a smaller cell body have shorter stem axons and may regulate the transmission of sensory signals as they pass the T junction. (b) DRG neuron soma are surrounded by satellite glia cells (SGCs). Neuronal soma in the DRG may communicate with each other through the formation of gap junctions between neurons or SCGs

DRG neurons are a heterogeneous population of cells and can be characterised based on a number of different parameters including soma size (Figure 4). DRG neuron somas range in diameter from ~20 to 100 μm and are generally categorised as either small (<25 μm), medium (25–35 μm) or large (>35 μm). This is directly related to axon myelination and hence conduction velocity. Neurons with a small diameter soma relate to unmyelinated C‐fibres (predominantly nociceptors, but also CLTMRs), medium‐sized neurons are thinly myelinated A‐δ nociceptors and Aδ‐LTMRs, whereas neurons with large cell bodies are thickly myelinated Aβ fibres or proprioceptors. The DRG also contains satellite glia cells (SGCs) which surround the cell bodies of primary sensory neurons. SGCs express receptors necessary to communicate with neurons and express certain ion channels which allow them to regulate the microenvironment surrounding neuronal soma (Vit et al., 2008). Although the exact physiological role of SGCs in the DRG is still unclear, the close contact of these two cell types indicates an important functional relationship, potentially performing a similar role to that of astrocytes in the CNS (Hanani & Spray, 2020).

As mentioned, DRG neurons have a unique structure; they are pseudounipolar meaning that they have a single axon (the stem axon) which leaves the cell body and bifurcates at the T junction, with one branch going towards its peripheral target and the other along the dorsal root into the CNS, and this set‐up is generally considered to allow for sensory information to travel uninterrupted along this route (Figure 4a). In some neurons, the proximal section of the stem axon can form a twisting glomerular structure which can substantially increase the distance between the soma and T junction (Matsuda et al., 2005). Both the cell soma and the stem axon are electrically excitable. This is postulated to prevent conduction block at the T junction since the soma and the stem axon will increase membrane capacitance and decrease membrane resistance, comprising spike propagation (Devor, 1999). Modelling systems propose that this is not the case for DRG neurons with large cell somas, as elimination of soma and stem axon excitability, had no impact on conduction (Amir & Devor, 2003). However, modulation of structural parameters, for example, by shortening the stem axon, impacts through conduction, suggesting that a greater stem length electrically isolates the soma preventing it from impacting on sensory transmission. Although to date no thorough analysis of stem axon length has been conducted, it is generally considered that the length is greater in DRG neurons with large cell soma versus smaller neurons (Matsuda & Uehara, 1981). Aligned with this, studies have suggested that the structural make‐up of this compartment in C‐fibres, that is a smaller soma and shorter stem axon, may allow some influence on through conduction and may serve as a low pass filter regulating high‐frequency input to the CNS (Du et al., 2014; Gemes et al., 2013; Sundt, Gamper & Jaffe, 2015). The excitability of the soma and stem axon does allow action potentials to invade the cell body (Amir & Devor, 2003) which has been demonstrated *in vivo* through recent calcium imaging experiments (Chisholm et al., 2018). The exact need for spike invasion into the soma is still not clear. It may simply be a way to regulate cell metabolism in line with sensory transmission, although other suggestions include a role in soma‐to‐soma communication at the level of the DRG (Devor, 1999). Although there are no synapses in the DRG, this neuron‐to‐neuron communication may be facilitated by the electrical coupling of somas through the formation of gap junctions (Spray & Hanani, 2019) (Figure 4b). This was first realised by Devor and Wall (Devor & Wall, 1990) and has also been shown in recent *in vivo* calcium imaging experiments (Kim et al., 2016), although this phenomenon seems more obvious under pathophysiological rather than physiological conditions. As well as this direct cross‐talk, the soma of DRG neurons can release neurotransmitters (Du et al., 2017; Kung et al., 2013; Zhang et al., 2007) and express the receptors to respond to such factors (Hanack et al., 2015; Kung et al., 2013), which may regulate how the soma and stem axon influence the transmission of sensory signals. For example, recent data show that GABA signalling in the DRG can lead to an increase in membrane conductance and depolarisation of the T junction (due to high intracellular Cl‐ in DRG neurons), preventing through conduction in nociceptive neurons, which is supported by the fact that local GABA application in the DRG reduces pain behaviour induced by chemical algogens applied to the paw (Du et al., 2017). While the pseudounipolar structure of DRG neurons means that the cell soma and stem axon are removed from the line of action potential propagation, rather than being passive, these structures may well impact on sensory transmission and hence regulate pain sensitivity.

### 4.2 Structural changes in the DRG following nerve injury or disease

In the context of nerve injury, there is evidence of fundamental anatomical changes in the DRG and for this structure being an important site in the generation of ectopic activity and contributing to neuropathic pain. One of the most profound changes suggested to occur in the DRG following nerve injury is that of soma loss due to neuronal cell death. In rodent models following damage to either the sciatic nerve or related spinal branches, a 20–40% loss of neuronal cell bodies has been documented, occurring over a 1–8 week period depending on the nature and degree of injury (Groves et al., 1997; Himes & Tessler, 1989; McKay Hart et al., 2002). This is coupled with a significant reduction in DRG volume likely due to the reduced cellular composition which also includes death of SGCs (McKay Hart et al., 2002; West et al., 2007). These remarkable changes have been somewhat debated, but a recent approach using an automated stereological platform and tissue clearing of whole mouse DRG support these findings, reporting a loss of ~37% of neurons in the L4 DRG 6 weeks post‐SNI (West et al., 2019). These findings are relevant to the clinical setting, with evidence suggesting that loss of DRG neuron soma also happens in patients following peripheral nerve injury (West et al., 2013). Mechanistically, loss of trophic support is an important factor. For example, nerve repair immediately following transection or the use of exogenous neurotrophic factors can prevent injury‐induced loss of DRG neurons (Groves et al., 1999; Ljungberg et al., 1999), and cell death seems to occur via apoptosis, due to the observation of DNA fragmentation and expression of proteins, such as caspase‐3 (McKay Hart et al., 2002; Wiberg et al., 2018). It seems reasonable that this loss could contribute to loss of sensation which accompanies nerve damage, however, it might also contribute to neuropathic pain. Injuries which result in a greater loss of neurons also result in more persistent pain following nerve damage (Sekiguchi et al., 2009) and treatment with neurotrophic factors, for example, Glial derived neurotrophic factor (Gdnf), which may prevent neuronal loss can attenuate neuropathic pain in animal models (Boucher et al., 2000). Furthermore, if this loss occurred in specific DRG neuron populations, for example those which may reduce nociceptive transmission through soma to soma communication (Du et al., 2017), this may also provide an explanation as to why soma loss leads to increased pain sensitivity. Interestingly, non‐peptidergic neurons (which are the target of Gdnf), have greater activation of apoptotic pathways following nerve damage (Wiberg, Novikova & Kingham, 2018), suggesting that certain DRG neuron sub‐populations may well be more susceptible to injury‐induced cell death.

Although there may be a loss of some neuronal cell bodies from the DRG following injury, the majority remain and there is evidence of increased communication between cell somas. When studying soma activity following nerve injury, Devor and Wall noticed cross‐excitation of neighbouring neurons (Devor & Wall, 1990). This was initially proposed to be mediated chemically (Amir & Devor, 1996), however, another possibility is one of more direct communication through the coupling of neuron‐glia units within the DRG. This coupling is thought to be enhanced following nerve injury as evidenced by the transfer of membrane impermeable dyes and the increase in linked activation of adjacent neurons visualised using *in vivo* calcium imaging (Hanani et al., 2002; Kim et al., 2016). This coupling occurs through the formation of gap junctions which are made up of connexin sub‐units that allow the transfer of ions and other molecules between cells and accordingly connexins are upregulated in sensory ganglia following nerve injury (Kim et al., 2016; Ohara et al., 2008). This data indicate that significant structural connectivity changes occur in the DRG following nerve injury that may contribute to neuropathic pain. These coupling events can occur between the same or different neuronal subtypes and may represent mechanisms contributing to hyperalgesia (nociceptors recruiting more nociceptors) or allodynia (LTMRS recruiting nociceptors). Moreover, there is evidence that these changes contribute to neuropathic pain. For example, blocking the action of gap junctions using either pharmacological or genetic approaches reduces coupled activation of neurons in the DRG and attenuates neuropathic pain in animal models of nerve injury (Kim et al., 2016; Ohara et al., 2008). Another anatomical change impacting neuron soma connectivity following nerve injury is that of aberrant sympathetic nerve sprouting in the DRG. These fibres which are normally only associated with blood vessels in the DRG, form basket‐like structures around the cell bodies of DRG neurons (particularly those with large diameters) following nerve injury (McLachlan et al., 1993). These structures are also seen in the DRG of patients with neuropathic pain (Shinder et al., 1999) and have been shown to impact the function of sensory neurons, with increased DRG soma excitability following signalling from the sympathetic fibres (Xie et al., 2010). Surgical, chemical or genetic interventions, which reduce sympathetic activity or the formation of these structures around DRG neuron soma, reduce neuropathic pain in animal models (Sun Ho Kim et al., 1993; Minett et al., 2014), although others have suggested a lack of sympathetic involvement in neuropathic pain (Ringkamp et al., 1999). Neuropathic pain is characterised by spontaneous or ongoing pain which is thought to result from ectopic activity of DRG neurons. It is of note that animal studies have indicated DRG soma as a key neuronal compartment for ectopic activity (Kajander & Bennett, 1992; Ma & LaMotte, 2007) and when activity is blocked here it can relieve neuropathic pain (Koplovitch & Devor, 2018; Weir et al., 2017). This is true in humans (Vaso et al 2014) and disrupting electrical activity at the level of the DRG soma and stem axon can selectively block activity in nociceptive afferents and can be used as a treatment for neuropathic pain in patients (Chao et al., 2020; Esposito et al., 2019).

Therefore, data suggest that the soma and stem axon of DRG neurons are important structures for sensory function. There is a fine balance as to how this compartment influences sensory propagation (Al‐Basha & Prescott, 2019) and therefore structural and connectivity alterations to the soma or stem axon following nerve injury may play an important role in neuropathic pain.

## 5 CENTRAL PROJECTIONS OF PRIMARY AFFERENTS

To transmit information from the periphery to the CNS, primary sensory neurons extend a central branch towards the spinal cord to synapse with second‐order neurons in the dorsal horn. Contrary to the organisation of peripheral axons, these central terminals convey input in a highly organised manner, with axons arranged according to their modality and sensory information converging onto a rich neuronal population in the spinal cord (Das Gupta et al., 2021; Gatto et al., 2021; Kuehn et al., 2019; Peirs et al., 2021). In this section, we will discuss the organisation of central projections in the spinal cord and highlight some of the structural changes which occur in the context of nerve injury and neuropathic pain.

### 5.1 Structure and function

**FIGURE 5:**
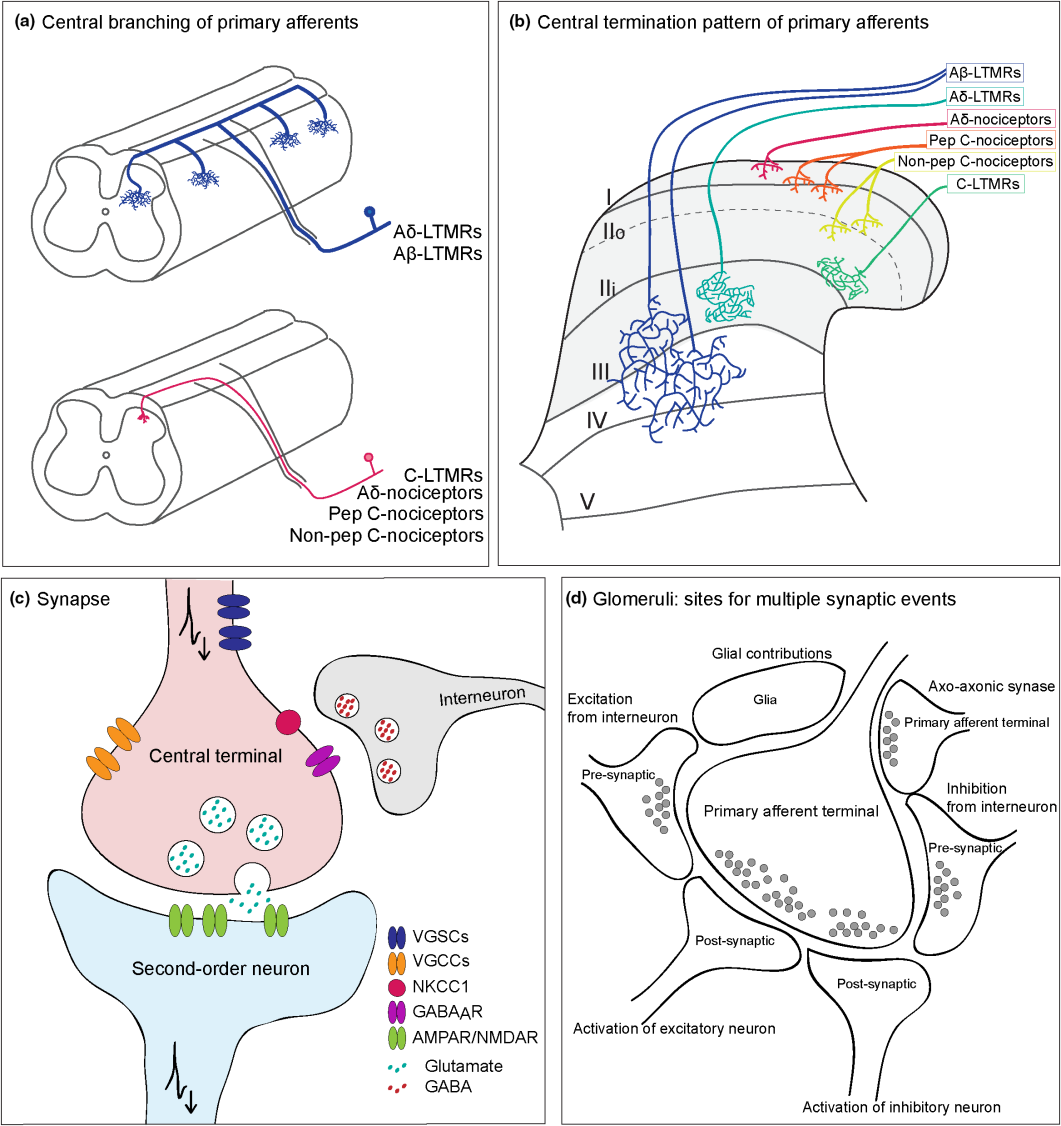
Unique features of central terminals of primary sensory neurons. (a) Sensory neurons exhibit distinctive branching patterns upon entering the CNS. Axons from Aβ afferents (blue) show a clear bifurcation, with branches extending rostrally and caudally over several segments and collaterals arising at regular intervals before growing deep into the spinal cord. In contrast, axons from Aδ and C‐fibres turn in one direction and reach the most superficial regions of the spinal cord. (b) Input from primary sensory neurons is organised in a distinct laminar manner. It is generally considered that nociceptive Aδ and C terminals are located in lamina I and II of the dorsal horn, while Aβ terminals from innocuous fibres are located deeper in laminae III–V. (c) Central terminals constitute the first synaptic relay of information from the activation of peripheral receptors to neurons in the dorsal horn. Sensory neurons release glutamate upon invasion of action potentials at central terminals through a VGCC‐dependent mechanism and vesicle fusion to the plasma membrane. This synapse is highly regulated by pre‐ and post‐synaptic mechanisms, including PAD from GABAergic interneurons. (d) Glomerular complexes enable central axons to engage with a large number of neurons and glial cells. While most central terminals form simple synaptic arrangements (axo‐somatic/axo‐dendritic), glomeruli allow interaction of central axons with several pre‐ and post‐synaptic structures. This enables the transmission of sensory information to many post‐synaptic targets but also the pre‐synaptic modulation of afferent input

Past the DRG, axons of primary sensory neurons bundle together forming the dorsal roots (DRs) and enter the spinal cord at the dorsal root entry zone (DREZ). The crossing of the DREZ occurs during early embryonic stages while sensory neurons carry the intrinsic capacity to project accurately to their distinctive targets in the spinal cord (Marmigère & Ernfors, 2007). Primary sensory axons exhibit unique branching patterns. Typically, axons from Aβ fibres bifurcate into ascending and descending arms, with collaterals arising at regular intervals along with several segments before diving deep into the grey matter of the spinal cord (Abraira & Ginty, 2013; Lucas‐Osma et al., 2018) (Figure 5a). On the other hand, axons associated with Aδ and C‐fibres do not bifurcate at the DREZ, but turn in one direction before immediately projecting towards the grey matter of the spinal cord (Li et al., 2011; Olson et al., 2017; Zylka, Rice & Anderson, 2005). Axon branching often determines how neurons relay information to their postsynaptic targets, not only as a means to reach several targets but also since branch points influence the conduction of actions potentials along with the fibre (Debanne et al., 2011; Kaczmarek & Jankowska, 2018; Li et al., 2020; Sundt, Gamper & Jaffe, 2015). In particular, bifurcation of myelinated primary sensory axons determines the extension of their termination fields in the spinal cord, and absence of bifurcation, due to altered cGMP signalling, leads to impaired processing of somatosensory information (Schmidt et al., 2016; Troster et al., 2018). Interestingly, axon branching also determines how axons regenerate after a lesion, according to the position of the lesion with respect to a branch point (Lorenzana et al., 2015).

At the spinal cord, sensory afferents converge onto a rich and heterogeneous population of neurons, and sensory input is determined by the specific distribution of axon terminals and their synaptic connections throughout the grey matter (Figure 5b). Central terminals of skin‐innervating sensory neurons are mostly confined to the dorsal horn, which is formed by the ensemble of laminae I–VI, as defined by Bror Rexed (Rexed, 1952). The organisation of primary afferents in the dorsal horn has been studied extensively, and they have been found to terminate in distinct laminae, according to their specific functions or peripheral targets (Abraira & Ginty, 2013; Abraira et al., 2017; Emery & Ernfors, 2020; Lallemend & Ernfors, 2012; Li et al., 2011; Todd, 2010). For example, Aδ and C‐fibre nociceptors terminate in laminae I and II; specifically, Aδ nociceptors and C peptidergic fibres terminate in lamina I and outer lamina II (IIo), while non‐peptidergic C‐fibres terminate predominantly in inner lamina II (IIi) (Choi et al., 2020; Ferrini et al., 2020; Pinto et al., 2008; Todd, 2010). Lamina I is one of the main hubs for projection neurons in the spinal cord, which suggests that nociceptive input to these superficial laminae could be immediately relayed to supraspinal pain centres (Choi et al., 2020; Cordero‐Erausquin et al., 2009; Ferrini et al., 2020). Indeed, nociceptive circuits in lamina I seem to be more sensitive to incoming input and readily undergo plasticity, which may help the system detect high‐threshold input to minimise damage (Ferrini et al., 2020). On the other hand, LTMRs, responsible for innocuous mechanical sensation terminate in deeper laminae: C‐LTMRs terminate in inner LII, close to the border with LIII (Larsson & Broman, 2019; Salio et al., 2020; Seal et al., 2009), while Aδ mechanoreceptors and Aβ fibres terminate in III–V in a largely overlapping manner (Abraira et al., 2017; Kuehn et al., 2019; Li et al., 2011). Another subset of projection neurons is found in lamina V, where input from innocuous Aβ‐ and nociceptive Aδ/C‐fibres converge into wide dynamic range (WDR) neurons, which are important for somatosensory processing in the spinal cord (Basbaum et al., 2009; Craig, 2003). The apparent segregation in the organisation of dorsal horn circuits, particularly regarding nociceptive input, which targets the most superficial laminae, suggests a linear coding of specific sensations. However, the segregation of labelled lines is maintained by a rather heterogeneous neuronal population in the dorsal horn, and processing of specific sensations must also account for their specific interconnections and interactions (Prescott, Ma & De Koninck, 2014). Processing by the CNS will decode sensory information into corresponding sensations, but this ultimately depends on how information is represented by primary afferents themselves (Chisholm et al., 2018; Wang et al., 2018).

To convey information to the spinal cord, central projections of sensory neurons are enriched with voltage‐gated ion channels, which enable the transmission of action potentials to the presynaptic terminal (Figure 5c). While the expression of ion channels may be similar in all axon regions, little is known about the specific subtypes of Nav channels expressed at central terminals (Bennett et al., 2019; Goodwin & McMahon, 2021). Yet, the expression of Nav1.7 in nociceptive terminals seems to be critical for neurotransmitter release, and loss of Nav1.7 in central terminals leads to severe reductions in synaptic transmission (MacDonald et al., 2021). Clusters of sodium channels are mostly located at major branch points, and are often absent from terminal branches and boutons, which helps propagation of action to small branches (Lucas‐Osma et al., 2018). Importantly, when axons cross into the CNS, central branches of A‐fibres are no longer myelinated by Schwann cells but by oligodendrocytes. Myelinating oligodendrocytes are necessary for the clustering of sodium channels as well as for regulating clustering of Caspr along the axon (Eisenbach et al., 2009; Kaplan et al., 1997). The presence of multiple branch points as well as myelination by different glial cells may also account for the slower conduction velocities in the central branch compared to the peripheral branch (Luscher & Shiner, 1990; Waddell et al., 1989). Once action potentials invade central terminals, they activate presynaptic calcium channels that determine vesicle fusion and neurotransmitter release.

As is the case with other primary afferents, cutaneous sensory neurons are glutamatergic and thus form excitatory synapses with neurons in the dorsal horn. Most of these synapses are simple axo‐dendritic or axo‐somatic, but others occur in complex synaptic arrangements termed glomeruli (Figure 5d). A single glomerular terminal may engage with multiple neurons and glial cells, which enables the transmission of sensory information to several postsynaptic targets (Ribeiro‐da‐Silva & De Koninck, 2008). Within the glomerular synaptic structure, central terminals of primary afferents receive axo‐axonic contacts from local interneurons to modulate transmission of sensory information (Boyle et al., 2019; Hughes et al., 2012). In fact, axo‐axonic contacts, typically from GABAergic neurons, are a powerful means to modulate synaptic transmission and are considered the structural basis for presynaptic inhibition (Rudomin & Schmidt, 1999). Activation of GABA_A_ receptors at presynaptic terminals has a depolarising effect due to the high intracellular concentration of chloride in sensory neurons, mainly due to the activity of a chloride cotransporter, NKCC1 (Mao et al., 2012; Plotkin et al., 1997; Sung et al., 2000). Despite being depolarising, primary afferent depolarisation (PAD) decreases the strength of action potentials invading presynaptic terminals, due to shunting or by inactivation of Na^+^ and Ca^2+^ channels (Graham & Redman, 1994). AMPA and NMDA glutamate receptors are also expressed in central terminals and may produce PAD of A‐ and C‐fibres, thereby also contributing to presynaptic inhibition (Bardoni et al., 2004; Lee et al., 2002; Marvizon et al., 2002). Interestingly, presynaptic inhibition produced by GABA or by glutamate seems to be distinct, where activation of LTMRs produce presynaptic inhibition by GABA_A_ receptors onto similar afferent subtypes, whereas C‐fibres produce presynaptic inhibition in an NMDA‐receptor‐dependent manner that inhibits large fibres, such as Aβ fibres (Zimmerman et al., 2019). Moreover, C‐fibres often found forming glomerular complexes may optimise the modulation of these fibres by different presynaptic mechanisms (Ribeiro‐Da‐Silva, 2004). Indeed, monosynaptic input from nociceptive C‐fibres is modulated by presynaptic inhibition through the activation of both low‐ and high‐threshold afferents, an important mechanism for the processing of nociceptive input to the spinal cord (Fernandes et al., 2020; Witschi et al., 2011).

The synapse formed between central terminals of primary afferents and second‐order neurons in the dorsal horn constitutes an important relay point for the transmission of sensory information to the CNS. While modulation of this information or transfer between neurons may occur prior to this point (see section 3), regulation of the central terminal is of critical importance as information is integrated and conveyed along the somatosensory pathway. Thus, alterations at this site, which may occur in pathological conditions, will ultimately affect how painful information reaches the brain.

### 5.2 Structural changes in central afferents following injury or disease

After nerve injury, anatomical changes include degeneration or retraction of central terminals away from the spinal cord, thereby interrupting synaptic transmission to the CNS. Loss of peptidergic and non‐peptidergic innervation in the superficial dorsal horn has been suggested in an animal model of HIV drug‐induced neuropathy, where a loss of IB4 and CGRP staining in the dorsal horn of the spinal cord has been associated with mechanical and thermal hyperalgesia (Huang et al., 2013). A similar loss of central terminals occurs after transection of the peripheral nerve, but not after crush nerve injury, where only a transient degeneration of non‐peptidergic afferents and mechanical hyperalgesia is observed (Bailey & Ribeiro‐da‐Silva, 2006; Casals‐Diaz et al, 2009). These findings highlight differences in the contribution of peptidergic and non‐peptidergic sub‐populations and changes to their central terminals in the development and maintenance of neuropathic pain. Contrary to peripheral axons, which maintain some regeneration potential after injury, central axons lack the substrates for proper axonal regrowth and guidance. Moreover, the DREZ acts as a barrier, preventing the access of regenerating axons into the spinal cord. This is due in part because, throughout development, the CNS acquires a rich extracellular matrix (ECM), with high levels of chondroitin surface proteoglycans (CSPG), which inhibit spontaneous recovery, limiting axon regeneration across the DREZ (Steinmetz et al., 2005). After nerve injury, there is some regeneration of axons, which are then unable to cross into the CNS (Mar et al., 2016). Indeed, changes in ECM proteins are factors that contribute to inflammatory and chronic pain aetiologies (Tansley et al., 2018; Parisien et al., 2019). Similar to peripheral axons, ongoing neuronal activity greatly improves the regenerative capacity of central afferents, especially in concert with the dissolution of ECM proteins (Steinmetz et al., 2005; Wu et al., 2020). However, even when axons are finally lured into the spinal cord, their distribution does not replicate the exact pattern prior to injury and some ectopic axon projections can be formed, meaning these structural changes no longer reflect the modular pattern of afferent terminations found in the naive spinal cord (Lekan et al., 1996; Okamoto et al., 2001). In particular, it has been suggested that nerve injury may produce reorganisation of Aβ fibres, which sprout from deeper laminae into more superficial layers and make functional synaptic contacts, a phenomenon that may underlie allodynia (Woolf et al., 1992). However, nociceptors with terminal arborisations that resemble Aβ fibres seem to account for the fibres innervating superficial laminae after injury, especially since the termination pattern of myelinated fibres is not changed (Shehab et al., 2003; Woodbury et al., 2008). Furthermore, Aβ fibres do not recover normal physiological properties even after they are able to regenerate into the spinal cord, probably due to aberrant myelination after injury (Tan et al., 2007). C‐fibres also undergo significant structural rearrangements. After peripheral nerve injury, there is an increase in synaptic markers mostly associated with C‐fibres, which may regulate stimulus‐evoked synaptic transmission in pain states (Sun et al., 2006). These fibres also form large varicosities enriched with calcium channel containing α2δ sub‐units, which may increase convergence of nociceptive signals in the spinal cord (Li et al., 2004, 2014; Yamanaka et al., 2021).

Increased spontaneous activity in primary afferents is another important hallmark of neuropathic pain in rodents (Haroutounian et al., 2014; Khan et al., 2002; Xiao & Bennett, 2008a, 2008b) and humans (Bostock et al., 2005; Ochoa et al., 2005; Orstavik & Jorum, 2010). Excessive input from primary sensory neurons helps initiate and maintain central sensitisation, which is the result of synapse strengthening by pre‐ and post‐synaptic mechanisms (Ikeda et al., 2006, 2007; Woolf & Salter, 2000). Following inflammation or nerve injury, increased activity at sensory neurons may lead to synaptic facilitation (Sandkühler & Gruber‐Schoffnegger, 2012). At central terminals, there is an increase in α2δ sub‐units of voltage‐gated calcium channels, which increase their density at the membrane, resulting in more neurotransmitter release (Ohnami et al., 2011). This enhanced synaptic transmission enables activation of normally silent NMDA glutamate receptors (NMDAR) at post‐synaptic neurons, thereby increasing post‐synaptic calcium (Ca^2+^) levels, along with Ca^2+^‐dependent signalling pathways. This cascade of events will also increase the excitability of the output neuron and facilitate the transmission of nociceptive information to the CNS (von Hehn et al., 2012; Latremoliere & Woolf, 2009).

Changes in PAD may underlie increased sensory neuron excitability after nerve injury. For example, an increase in expression and activity of NKCC1, which leads to further intracellular chloride accumulation in sensory neurons and a more depolarised chloride reversal potential (Pieraut et al., 2007), means that activation of GABA_A_ receptors may produce larger depolarisation leading to the generation of antidromic spikes, known as dorsal root reflexes, and further excitation of sensory neurons (Cervero et al., 2003; Laird et al., 2004; Willis Jr., 1999). In inflammatory pain conditions, such an increase in NKCC1 has been observed (Funk et al., 2008; Morales‐Aza et al., 2004), as well as following trigeminal nerve injury, with associated mechanical hyperalgesia (Wei et al., 2013). Yet, changes in chloride homoeostasis must be accompanied by intrinsic hyperexcitability of primary sensory neurons for the activation of GABA_A_ receptors to become excitatory, since a strong GABAergic conductance in sensory neurons is still capable of producing presynaptic inhibition at more depolarised chloride reversal potentials (Takkala, et al, 2016). Interestingly, after nerve injury, there is also a decrease in inhibitory terminals in the spinal cord as well as a decreased expression of GABA_A_ receptors in nociceptive fibres, which lead to mechanical and thermal hyperalgesia (Chen et al., 2014; Lorenzo et al., 2014; Meisner et al., 2010; Moore et al., 2002). Indeed, restoring inhibitory circuitry in the dorsal horn, using GABAergic cell transplantation, can reverse the hypersensitivity observed in models of both traumatic‐ and chemotherapy‐induced nerve injury (Bráz et al., 2012, 2015). Taken together, these results suggest that enhancing GABAergic tone may be a potential strategy to enhance presynaptic inhibition, especially at central terminals, which are ideally placed to relay information to the CNS (Weir et al., 2017).

## 6 SUMMARY

Primary sensory neurons are a diverse set of cells, able to convey information relating to a variety of specific sensations including touch and pain. Their heterogeneity can be defined based on an increasing number of molecular markers, but also by their anatomical distribution, structure and the organisation of specialised neuronal compartments. In terms of sensory endings in the skin, the node of Ranvier or central terminals in the spinal cord, we know that these compartments are important for detecting, conveying and transmitting sensory information and studies continue to increase our knowledge and appreciation of how these structures relate to sensory function, which is important in order to generate a more complete understanding of sensory neuron physiology. For other compartments, however, such as the cell soma, their influence on sensory transmission is less clear, but increasing evidence suggests that sensory neuron soma can directly communicate at the level of the DRG and, despite the pseudounipolar structure of sensory neurons, potentially modulate through conduction. The physical integrity of these compartments is key to their proper function. Nerve damage leads to structural changes which are commonly associated with neuropathic pain. This includes loss of fibres in the skin, nodal disorganisation, soma loss in the DRG and retraction of central terminals. Some of these changes are useful for diagnostic purposes, for example skin is easily accessible in patients for the analysis of nerve fibre density and nodal changes. Structural changes to these compartments lead to dysfunction and therefore may more directly contribute to neuropathic pain. Importantly, treatments which attenuate neuropathic pain in both patients and animal models are associated with normalising these changes. As we continue to improve our knowledge of the overall physiology of sensory neurons, it will remain important to consider their discrete compartments and whether experimental or therapeutic manipulation can be targeted to these specific regions, to gain a better understanding of their role in sensory physiology, offering more targeted interventions for painful conditions such as neuropathic pain.
